# TRAUMA-INFORMED PERSPECTIVES AND PRACTICES FOR TEACHING AND LEARNING IN GERONTOLOGY

**DOI:** 10.1093/geroni/igae098.0174

**Published:** 2024-12-31

**Authors:** Jennifer Sasser

**Affiliations:** Portland Community College, PORTLAND, Oregon, United States

## Abstract

With the increasing incidence of individual and shared traumatic events, as well as the complexity and poignancy of many of the issues considered in social gerontology and aging-related courses, there is a concomitant need for expanded awareness of the ways in which trauma shows up, both for our students as well as for ourselves as educators. What’s more, for students preparing for careers in the field of aging, especially those who intend to work directly with older people and their families, developing a trauma-informed disposition and skill-set is particularly important. In this interactive session, an ongoing initiative in the context of a community college applied social gerontology program to infuse the curriculum with trauma-informed perspectives will be shared. In addition, a framework for trauma-informed andragogical practices for teaching and learning about aging issues will be introduced. Enacting a trauma-informed approach, the presenter will facilitate an opportunity for participants to think together about ways to apply this powerful approach in the context of their own professional practices.

